# The PAX3-FOXO1 Fusion Protein Present in Rhabdomyosarcoma Interferes with Normal FOXO Activity and the TGF-β Pathway

**DOI:** 10.1371/journal.pone.0121474

**Published:** 2015-03-25

**Authors:** Michel Schmitt-Ney, Giovanni Camussi

**Affiliations:** Molecular Biotechnology Center and Department of Medical Sciences, University of Torino, Torino, Italy; Ospedale Pediatrico Bambino Gesu', ITALY

## Abstract

PAX3-FOXO1 (PAX3-FKHR) is the fusion protein produced by the genomic translocation that characterizes the alveolar subtype of Rhabdomyosarcoma, a pediatric sarcoma with myogenic phenotype. PAX3-FOXO1 is an aberrant but functional transcription factor. It retains PAX3-DNA-binding activity and functionally overlaps PAX3 function while also disturbing it, in particular its role in myogenic differentiation. We herein show that PAX3-FOXO1 interferes with normal FOXO function. PAX3-FOXO1 affects FOXO-family member trans-activation capability and the FOXO-dependent TGF-β response. PAX3-FOXO1 may contribute to tumor formation by inhibiting the tumor suppressor activities which are characteristic of both FOXO family members and TGF-β pathways. The recognition of this mechanism raises new questions about how FOXO family members function.

## Introduction

Rhabdomyosarcoma (RMS) is a pediatric soft-tissue sarcoma with myogenic phenotype, and is the most common soft-tissue sarcoma in children, adolescents and young adults [1]. RMS can be broadly divided into two major histopathologic subtypes: alveolar (ARMS) and embryonal (ERMS). Most (75%) ARMS cases display recurrent chromosomal translocation that fuses two transcription factor-encoding genes together; the *PAX3* gene (in a minority of cases the *PAX7* gene) and the *FOXO1* gene [2]. All *PAX3/7-FOXO1* translocation-positive tumors express common chimera *PAX3/7-FOXO1* mRNA and protein (unlike the reciprocal *FOXO1-PAX3/7* translocation product) indicating that its expression is required for ARMS tumorigenesis.

PAX3-FOXO1 has been extensively studied (reviewed in [3,4]), and functional studies have confirmed its active role in ARMS generation. Its N-terminal encompasses the PAX3 DNA-binding domains (paired and homeo domain) and is fused to the C-terminal of FOXO1 which comprises the truncated FOXO1 DNA-binding domain and the FOXO1 C-terminal transcriptional transactivation domain. PAX3 (like PAX7) is implicated in muscle generation [5]. PAX3-FOXO1 retains PAX3-DNA-binding activity and is capable of initiating a transcription program which overlaps, but is distinct from, the program generated by PAX3. PAX3-FOXO1 can promote partial myogenic differentiation in certain cellular contexts [6,7], but is also able to interfere with it [8,9]. However, it is not yet clear how much the transforming activity of PAX3-FOXO1 depends on its potential to interfere with complete myogenic differentiation.

We have hypothesized that PAX3-FOXO1 may affect specific cellular functions which are controlled by FOXO1 in normal cells. In particular, the FOXO-dependent TGF-β response may be affected because the section of the FOXO1 protein sequence that has been shown to be sufficient for the interaction with SMADs (see below) is in part present in PAX3-FOXO1. The TGF-β pathway has been widely implicated in cancer [10] and members of the FOXO family have been shown to functionally contribute to this pathway. FOXOs can directly interact with TGF-β mediators, SMAD3 and SMAD4 [11], and co-regulate the expression of *p21CIP* and *p15INK4B* [11,12]. Screening performed in keratinocytes [13], has identified nine additional genes, besides *p21CIP* and *p15INK4B*, whose regulation by TGF-β is dependent on both FOXOs and SMADs.

We herein show that ARMS cells that express PAX3-FOXO1 respond poorly to TGF-β and that the inhibition of PAX3-FOXO1 expression in these cells is able to partially restore TGF-β responsiveness (p15INK4b becoming TGF-β—inducible). In the complementary approach, the ectopic expression of PAX3-FOXO1 in TGF-β responsive cells leads to part of the TGF-β transcriptional response being perturbed.

PAX3-FOXO1 disturbance extends beyond the FOXO-dependent TGF-β pathway. We show that PAX3-FOXO1 can directly interfere with the transcription activity of the FOXO family which is made up of 4 members (FOXO1, FOXO3, FOXO4 and FOXO6) that act as monomers and possess very similar DNA-binding specificity [14]. They show different, but overlapping, expression patterns where FOXO1, 3 and 4 are rather ubiquitous while FOXO6 expression is more restricted. FOXO1, FOXO3 or FOXO4 gene disruption in mice gives rise to large differences in phenotype, which shows that, despite their similarity, they each possess important functional specificity. Of a range of cellular functions, FOXOs have been shown to act as tumor suppressors [15], and, in fact, FOXO1, FOXO3 and FOXO4 show redundancy for this function. The simultaneous disruption of all of their six alleles is required for restricted tumor formation in mice [16,17]. PAX3-FOXO1 expression may be functionally equivalent to this simultaneous disruption, leaving the cells that express PAX3-FOXO1 without the FOXO family member activity that is necessary for efficient tumor suppression.

## Results

### ARMS cells show altered TGF-β response

SMADs and FOXOs cooperate in the transcription regulation of several genes [13]. In particular, the *p21CIP1* and *p15INK4B* growth inhibitor-encoding genes bear, in their promoter, a composite FOXO-SMAD DNA binding site on which a SMAD-FOXO protein complex binds [11]. We speculate that PAX3-FOXO1 may be able to interfere with the functional cooperation between FOXOs and SMADs.

If this is true, PAX3-FOXO1 expressing cells should show an aberrant FOXO-dependent TGF-β response. We challenged two ARMS cell lines that express PAX3-FOXO1 with TGF-β and looked at their cell growth and gene expression response. The ARMS cell line response was compared to the response obtained with the ERMS cell line RD18 [18]. This is a clonal derivative of the RD cell line which is known to respond to TGF-β with growth arrest [19].

**Fig 1:**
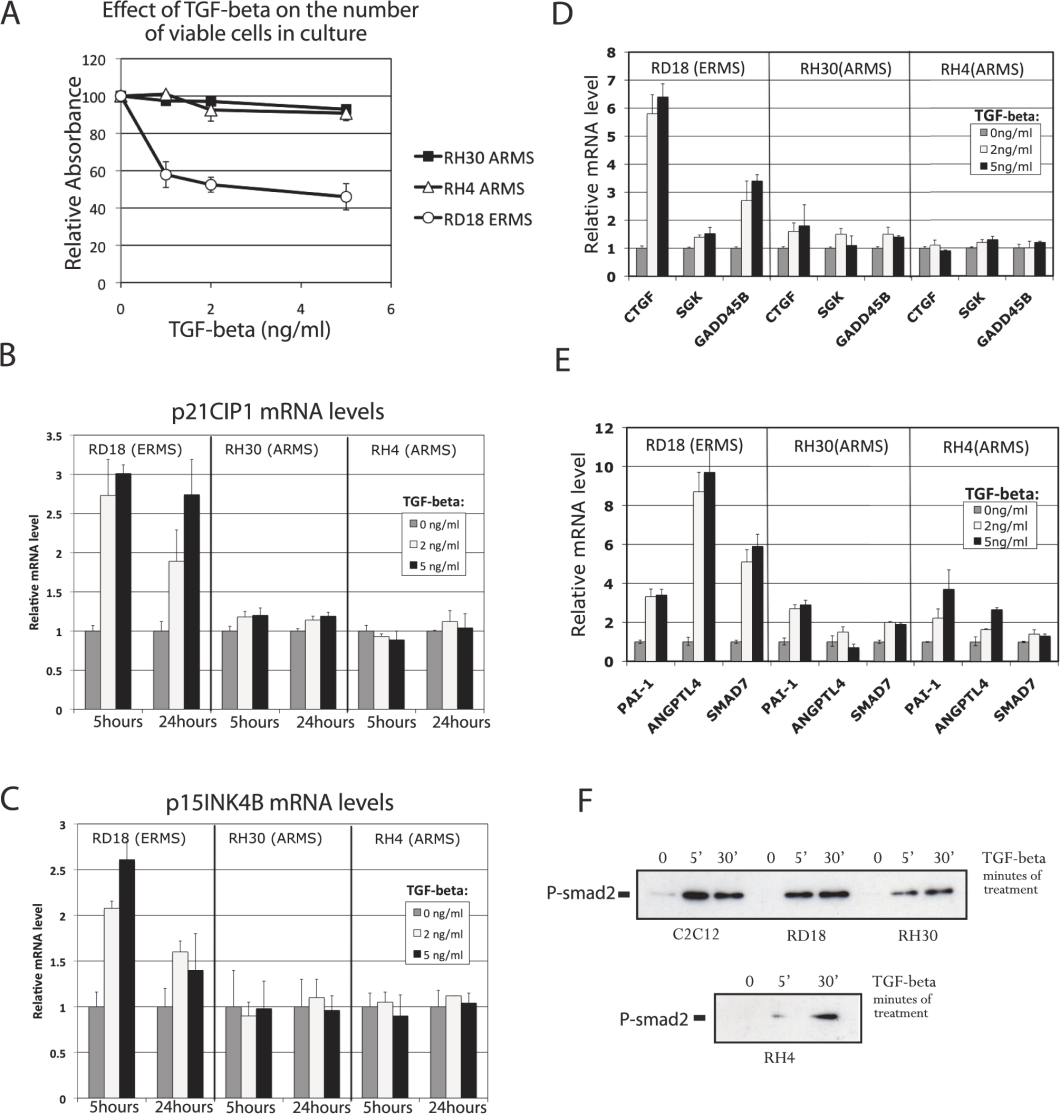
ARMS cell lines RH30 and RH4 are poor TGF-β responder. **(A)** The relative number of viable cells present in the culture after three days of TGFβ treatment (at the indicated concentrations) was evaluated using the colorimetric MTT assay (Millipore). (**B and C)** Relative levels of *p21CIP* and *p15INK4B* mRNAs were evaluated by real-time PCR after treatment with TGF-β. Concentrations of TGF-β and treatment times are indicated. **(D and E)** Relative mRNA levels of indicated target genes belonging to the FOXO-SMAD synexpression group [13] (**D**) and genes not belonging to the FOXO-SMAD synexpression group (**E**). Measurements were performed by real-time PCR after 5 hours of TGF-β treatment at the indicated concentration. **(F)** Phosphorylation levels of SMAD2 evaluated by western-blot using an anti-phospho-SMAD2 antibody. Cell lines and treatment times (in minutes) are indicated. All experiments were performed at least twice with the exception of panel F. Errors bars represent standard deviation of two experiments in panel A,B,C and three experiments in panel C, D and E.

As expected, ERMS cell line RD18, showed a strong growth inhibition response to TGF-β (confirming the results obtained by Ye et al. [19] on the TGF-β induced growth arrest of RD cells), while this response was not observed in ARMS cell lines, RH30 and RH4, which express the PAX3-FOXO1 translocation product (Fig. 1A).

We also investigated the transcriptional response to TGF-β treatment and selected representative genes which belong to three categories of known TGF-β-responsive genes: a- genes that possess a composite FOXO-SMAD binding site, in their promoter, that has been experimentally studied [11,12], cyclin-dependent kinase inhibitors *p21CIP* and *p15INK4B* (Fig. 1B and C); b- genes that belong to the FOXO-SMAD synexpression group (as determined in keratinocytes), but which do not possess a composite site (*CTGF*, *SGK*, *GADD45B*) (Fig. 1D); and c- genes that have not been reported to belong to the synexpression group (*ANGPTL4*, *SMAD7* and *PAI-1*) but are known transcriptional targets of TGFβ (Fig. 1E). Fig. 1B shows that the *p21CIP* gene is reproducibly induced by TGF-β in RD18 cells that are negative for *PAX3-FOXO1* expression. This regulation is not seen in PAX3-FOXO1 expressing ARMS cells. Results obtained with *p15INK4B* are very similar to those obtained with *p21CIP*, with reproducible induction by TGF-β in the RD18 cell line and no induction in the ARMS cell lines (Fig. 1C).

For the other genes analyzed, a generally weaker response was observed in the ARMS cells than in the ERMS cells (Fig. 1D and 1E) with the exception of *PAI-1* that showed a similar response in all cell lines.

In order to exclude the possibility that the different responses to TGF-β are due to a gross defect in the TGF-β pathway, we looked at the SMAD2 phosphorylation-status in response to TGF-β. The *PAX3-FOXO1*-positive ARMS cells, RH30 and RH4, demonstrated robust levels of SMAD2 phosphorylation upon TGF-β treatment (Fig. 1F), although it was a slightly weaker response than that observed in RD18 cells.

The results shown in Fig. 1 are consistent with our initial hypothesis but do not clearly demonstrate the role that PAX3-FOXO1 plays. The differences seen in the TGF-β response could merely reflect cell type specific variations in TGF-β response which are not related to the translocation event. The cells which give origin to ARMS have not yet been identified [20,21], and their TGF-β-response pattern might be similar to what is seen in ARMS cells. Alternatively, the long-term culture of cells may have affected their response to TGF-β.

### Inhibition of PAX3-FOXO1 expression in ARMS cells restores the response of *p15INK4B* to TGF-β and influences the basal expression of FOXO-regulated genes

**Fig 2:**
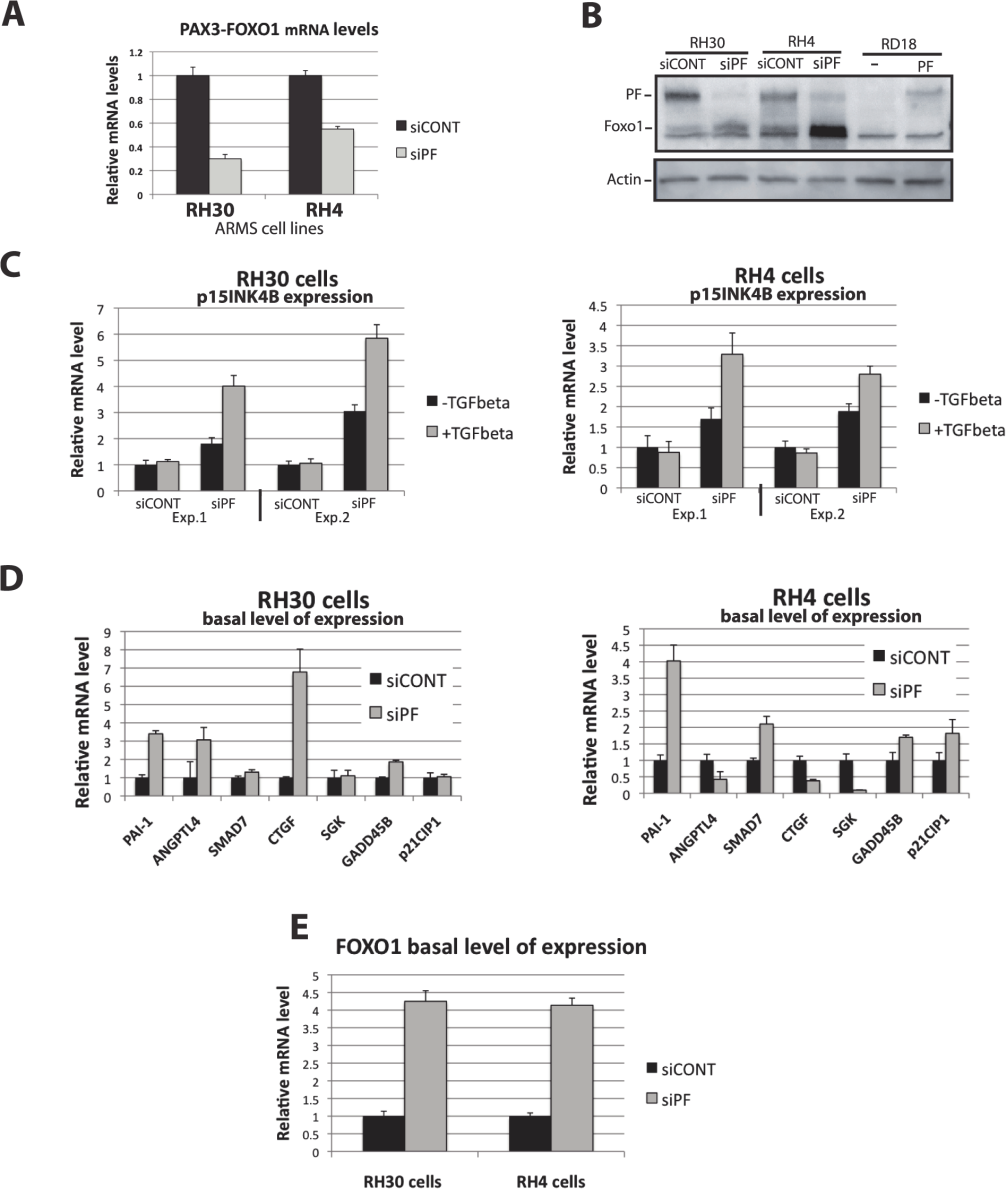
siRNA-mediated down regulation of PAX3-FOXO1 in two ARMS cell line restores p15INK4B -TGF-β-response and affects basal level of expression of FOXO-regulated genes. **(A and B**) siRNA against PAX3-FOXO1 (siPF) or control siRNA (siCONT) were transfected in RH30 and RH4 cell lines. 48 hours after transfection extracts were prepared. (**A**) Levels of *PAX3-FOXO1* mRNA were measured by real-time PCR. (**B**) Levels of PAX3-FOXO1 protein were evaluated by western blot (using an anti-FOXO1 antibody recognizing the C-terminal part of the FOXO1 protein present in PAX3-FOXO1). The position of FOXO1 and PAX3-FOXO1 are indicated. As a control for PAX3-FOXO1, extracts from RD18 cells or RD18 cells infected with a lentivirus carrying PAX3-FOXO1 expression were included. Actin levels were evaluated as loading control. **(C)** RH30 and RH4 cells were transfected with a control siRNA (siCON) or a siRNA targeting PAX3-FOXO1 (siPF), 32 hours (exp.1) or 48 hours (exp.2) post-transfection cells were challenged with TGF-β for 16 hours (Exp.1) or 7 hours (Exp.2) and *p15INK4B* mRNA levels were measured by real-time PCR. **(D and E)** Basal level of expression of indicated genes 40 hours after control (siCON) or PAX3-FOXO1-targetting siRNA (siPF) transfection in RH30 and RH4 cells as measured by real-time PCR. Errors bars represent standard deviation of three independent experiments except for panel C where they represent standard deviation of triplicates in single experiments.

In order to test whether the altered TGF-β-response in ARMS cells is due to the presence of PAX3-FOXO1, we challenged these cells with TGF-β after the siRNA-mediated inhibition of PAX3-FOXO1. The validated PAX3-FOXO1 breakpoint-specific siRNA (siPF) [22,23], and a control siRNA (siCONT) were transfected in RH30 and RH4 cells and then *PAX3-FOXO1* mRNA and protein levels were quantified. Efficient *PAX3-FOXO1* mRNA and protein level inhibition was observed after siPF-transfection (Fig. 2A and 2B). siRNA-transfected cells were then challenged with TGF-β. *p15INK4B* showed very reproducible recovery in TGF-β responsiveness in both ARMS cell lines following transfection with siPF, but not with the control siRNA siCONT (Fig. 2C). Importantly, the *p15INK4B* gene promoter bears a functional composite FBHE-SBE (Forkhead Binding Element-Smad Binding Element) located 518 bp upstream of the transcription initiation site that mediates FOXO-dependent TGF-β response [12]. Our results strongly indicate PAX3-FOXO1 interference on FOXO-SMAD.

Besides provoking *p15INK4B* gene TGF-β-responsiveness recovery, siPF also produced an increase in the *p15INK4B* basal expression level (Fig. 2C).

No convincing changes in TGF-β responsiveness were observed in any other gene upon siPF-transfection, with the exception of a slight effect seen in *GADD45B and CTGF* (data not shown). However, a robust and reproducible siPF-transfection effect was observed on the basal expression level of most of these genes (Fig. 2D). This unexpected basal expression effect has two possible explanations: a- relief from a block in autocrine-derived TGF-β response. Experiments using siRNA-mediated inhibition of PAX3-FOXO1 in the presence of the TGF-β inhibitor SB431542 do not support this possibility. However, the fact that SB431542 affected basal expression on its own made the interpretation of the data difficult (not shown); b- Relief from an inhibitory (or interfering) effect exerted by PAX3-FOXO1 on global cellular FOXO activity. This possibility prompted us to more carefully inspect the literature. We realized that two of the three genes that were selected because their regulation by TGF-β is not known to be dependent on FOXO (not part of the synexpression group), are, in fact, known to be under the effect of FOXOs. *PAI-1* is both indirectly [24,25] and directly regulated by FOXOs [26] and *ANGPTL4* is up-regulated by FOXO [27]. The promoter sequence of the third gene, *SMAD7*, was inspected and an element that closely matches the consensus FOXO binding site [28] was found. This sequence (TTGTTTGC) is conserved in human, mouse and rat SMAD7-gene promoters and is located 40 bp upstream of a function Smad Binding Element [29].

These findings further strengthen our suspicion that PAX3-FOXO1 may interfere with FOXO activity.

*FOXO1* gene expression is auto-regulated by FOXOs and in particular by FOXO3 [30,31,32]. *FOXO1* mRNA level measurements have been used as a read out for FOXO activity [33]. We therefore wondered whether PAX3-FOXO1 expression inhibition by siPF had an effect on *FOXO1* mRNA levels. Robust *FOXO1* mRNA up-regulation (Fig. 2E) was seen in both ARMS cell lines following siPF transfection. As a control, siPF had no effect on *FOXO1* mRNA levels in RD18 cells lacking PAX3-FOXO1 (data not shown), excluding a direct effect on *FOXO1* mRNA levels. The western in Fig. 2B confirms the above mRNA data. The antibody used recognizes the C-terminal part of FOXO1 and therefore is able to detect the PAX3-FOXO1 as well as the FOXO1 proteins. The western shows that there is an increase in FOXO1 protein levels after siPF transfection which is concomitant with the reduction in PAX3-FOXO1 protein levels.

### PAX3-FOXO1 inhibits FOXO activity on an IRS element

**Fig 3:**
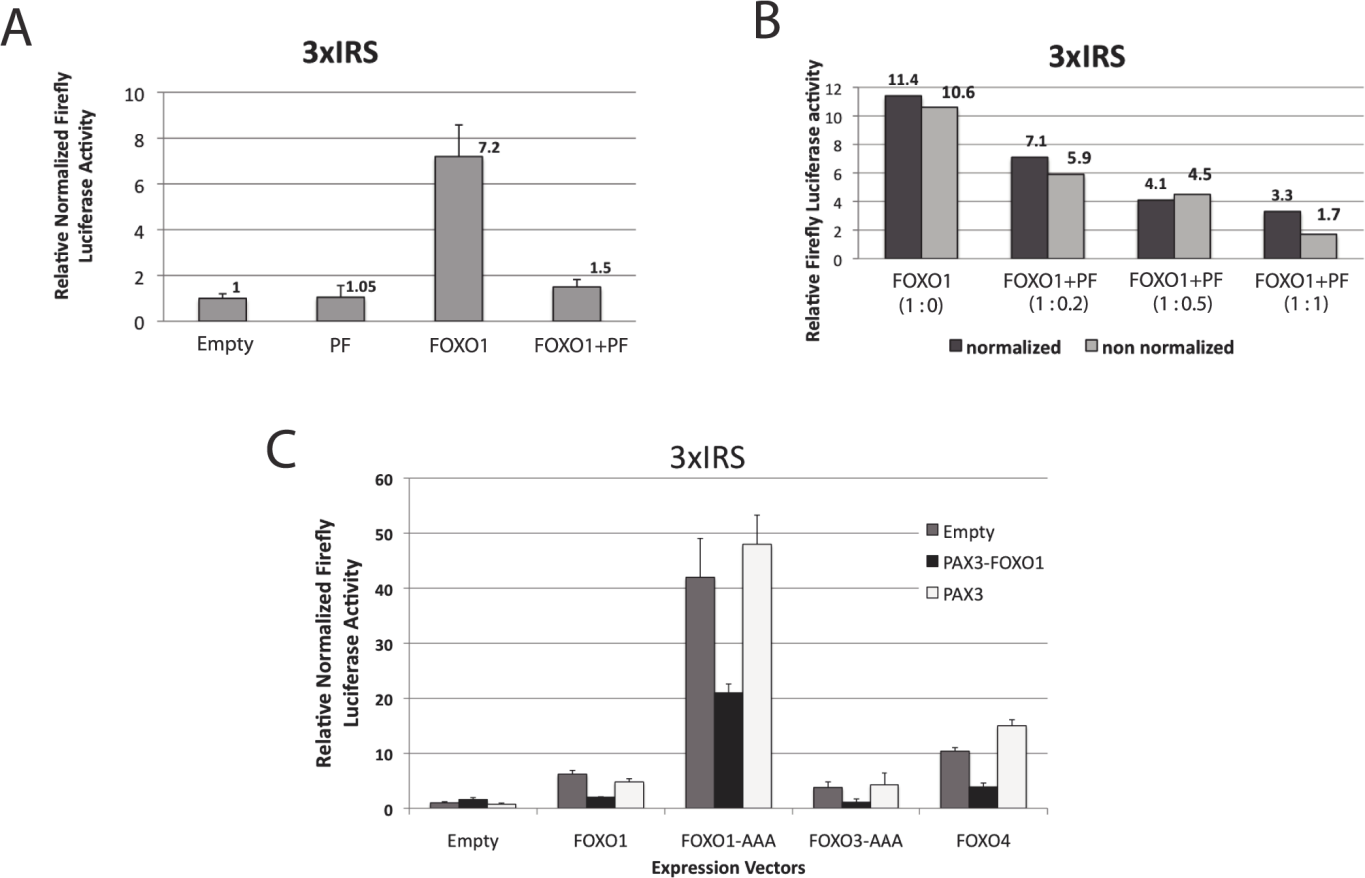
PAX3-FOXO1 inhibits FOXO-transactivation on a FOXO- response element (3XIRS). **(A, B and C)** 293T cells were co-transfected with a reporter plasmid (pGL2, promega) encoding for firefly luciferase under the control of a FOXO-responsive 3XIRS-containing promoter as well as expression plasmids encoding for the indicated protein (PF: PAX3-FOXO1). 48hours post-transfection, luciferase activity was determined. Where indicated a third plasmid encoding renilla luciferase was included for the control of transfection efficiency. **(B)** The relative proportion of the FOXO1 and PAX3-FOXO1 plasmids are indicated in brackets. The amount of FOXO1 plasmid was constant with increasing amount of PAX3-FOXO1 plasmid (total amount of transfected DNA was kept constant by using empty expression plasmid). **(C)** The indicated expression vectors were co-transfected with Empty, PAX3-FOXO1 or PAX3 encoding vectors and transactivation on the 3XIRS containing reporter was evaluated by firefly luciferase measurement normalized to co-transfected renilla luciferase activity. Errors bars represent standard deviation of three experiments.

The experiments shown in Fig. 3 address the question of PAX3-FOXO1's ability to interfere with FOXO function. 293T cells were transfected with a FOXO-responsive luciferase-reporter plasmid (3XIRS) that contains IGFBP-1 gene promoter sequences that are responsible for insulin repression. This sequence mediates transcription activation by FOXOs through the FHBE (forkhead binding element) that it contains [34]. Expression vectors that encode for FOXO1 and PAX3-FOXO1 were co-transfected both separately and together.

As expected, FOXO1 strongly transactivated the FHBE-containing promoter. PAX3-FOXO1 (PF) had no effect on the FHBE-containing reporter’s activity, which is expected for a protein that is unable to bind the FHBE DNA element. PAX3-FOXO1 clearly inhibited FOXO1 activity when both expression vectors were mixed. This effect was concentration dependent (Fig. 3B). We also tested whether the inhibition exerted by PAX3-FOXO1 extended to other FOXO family members. Fig. 3C shows that PAX3-FOXO1 was able to inhibit wild type or constitutively-active nuclear mutant forms (with threonine/serine to alanine mutations on three AKT phosphorylation sites) of FOXO1, FOXO3 and FOXO4. A control wild type PAX3 was not able to inhibit FOXO-transactivation in this assay.

### PAX3-FOXO1 inhibits TGF-β inducibility and SMAD transactivation on an FHBE-SBE composite element

**Fig 4:**
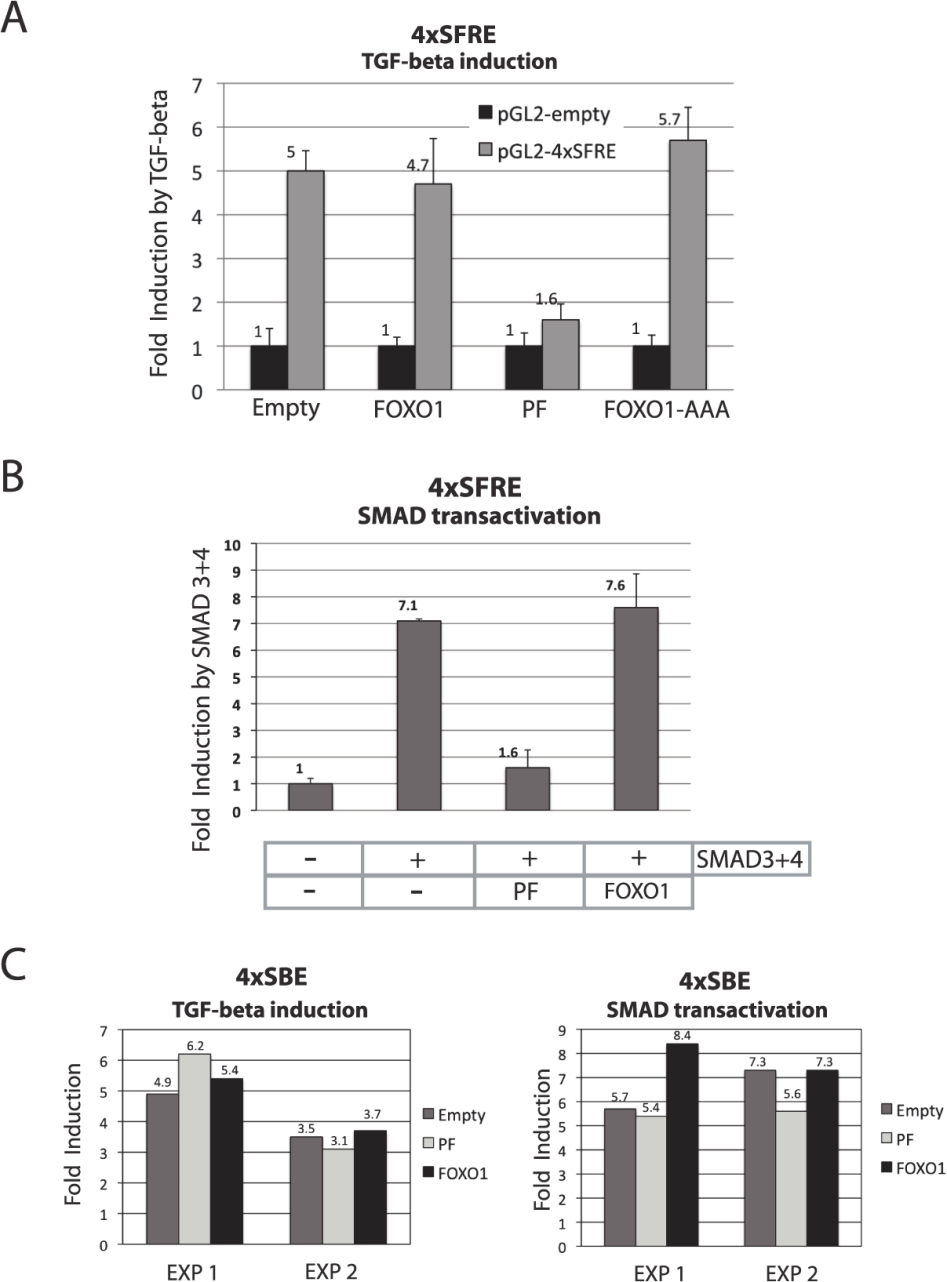
On a bipartite FOXO-SMAD responsive element (4XSFRE), PAX3-FOXO1 inhibits TGF- response and SMAD transactivation. **(A)** 293T cells were transfected with pGL2-promoter control reporter (pGL2) or a pGL2-promoter reporter containing a bipartite FOXO-SMAD response element (pGL2-4XSFRE) and the indicated expression plasmids (PF: PAX3-FOXO1). In order to avoid transfection efficiency interference, one well of individual transfected cells were trypsinized 24 hours after transfection and splitted in 6 wells. 8 hours later, three wells were left untreated the other three were treated with TGF- (5ng/ml) for further 16hours. Fold induction by TGF- is shown for each transfection. (**B)** 293T cells were transfected with a pGL2-promoter reporter containing a bipartite FOXO-SMAD response element (pGL2-4XSFRE) and the indicated expression plasmids (PF: PAX3-FOXO1) with or without SMAD3 and SMAD4 expression plasmids. Firefly luciferase activity was determined 48 hours post-transfection and normalized to co-transfected renilla luciferase for correcting for transfection efficiency. **(C)** 293T cells were transfected with a SMAD binding site-containing reporter, 4XSBE-LUC and the indicated expression plasmids (PF: PAX3-FOXO1). Left panel: TGF- induction was measure. The procedure to avoid transfection efficiency interference was the same as for panel **A**. Right panel: in addition to the indicated plasmids cells were cotransfected with or without SMAD3 and 4 and fold induction by SMADs was calculated like in panel **B**. Results of two independent experiments are shown. All experiments were performed at least three times except for panel **C**. Error bars represent standard deviation of three independent experiments.

The composite FOXO-SMAD responsive element (SFRE, Smad Foxo Response Element) present in the p21 gene promoter mediates FOXO-dependent TGF-β inducibility [11]. We evaluated whether this FOXO dependent TGF-β responsiveness is affected by PAX3-FOXO1 in a trans-activation assay. Fig. 4A clearly shows that this is the case. The robust induction seen with the SFRE-containing reporter (no induction is seen with the pGL2 control vector) after TGF-β treatment was compromised when PAX3-FOXO1 (PF) was co-transfected with the reporter vector. It is worth noting that FOXO1 did not increase the induction exerted by TGF-β. It is probable that endogenous levels of FOXOs are sufficient for full response. FOXO1-AAA (a constitutively nuclear-localized FOXO1 mutant that AKT is unable to phosphorylate) did not show any inhibitory effect when used as a control. This excludes the possibility that the inhibition exerted by PAX3-FOXO1 (which is also constitutively nuclear) is solely due to elevated expression of nuclear-localized FOXO coding sequences that may have provoked an artificial squelching effect via the exhaustion of cofactors.

In a different setting (Fig. 4B), we also observed strong SMAD-transactivation inhibition by PAX3-FOXO1 (PF), using SMAD3 and 4 over-expression instead of TGF-β treatment, which again was not observed with FOXO1 over-expression. This result is an independent confirmation of the result obtained in Fig. 4A.

As a control, we tested the effect of PAX3-FOXO1 expression on the FOXO-independent TGF-β response using a SMAD-responsive element (SBE: SMAD Binding Element) containing reporter plasmid. Fig. 4C shows that the induction seen on the SBE after TGF-β treatment or co-expression of SMAD3 and 4 was not affected by PAX3-FOXO1 co-expression. In fact, differently to observations on the 4XSFRE element, PAX3-FOXO1 and FOXO1 have a similar behavior on the SBE. It is also worth noting that for an unknown reason, both, PAX3-FOXO1 and FOXO1 exerted a strong and similar repressive effect on the basal activity of this reporter plasmid (data not shown).

Importantly, the results of Fig. 4C show that the PAX3-FOXO1 interference observed in Fig. 4A and B is a specific effect that requires the presence of composite FOXO-SMAD responsive element in the reporter.

Altogether these results corroborate findings on the recovery of *p15INK4b*-TGF-β-inducibility following PAX3-FOXO1 expression inhibition in ARMS cells and provide a hint on the possible mechanism involved.

### Effects of Ectopic expression of PAX3-FOXO1

**Fig 5:**
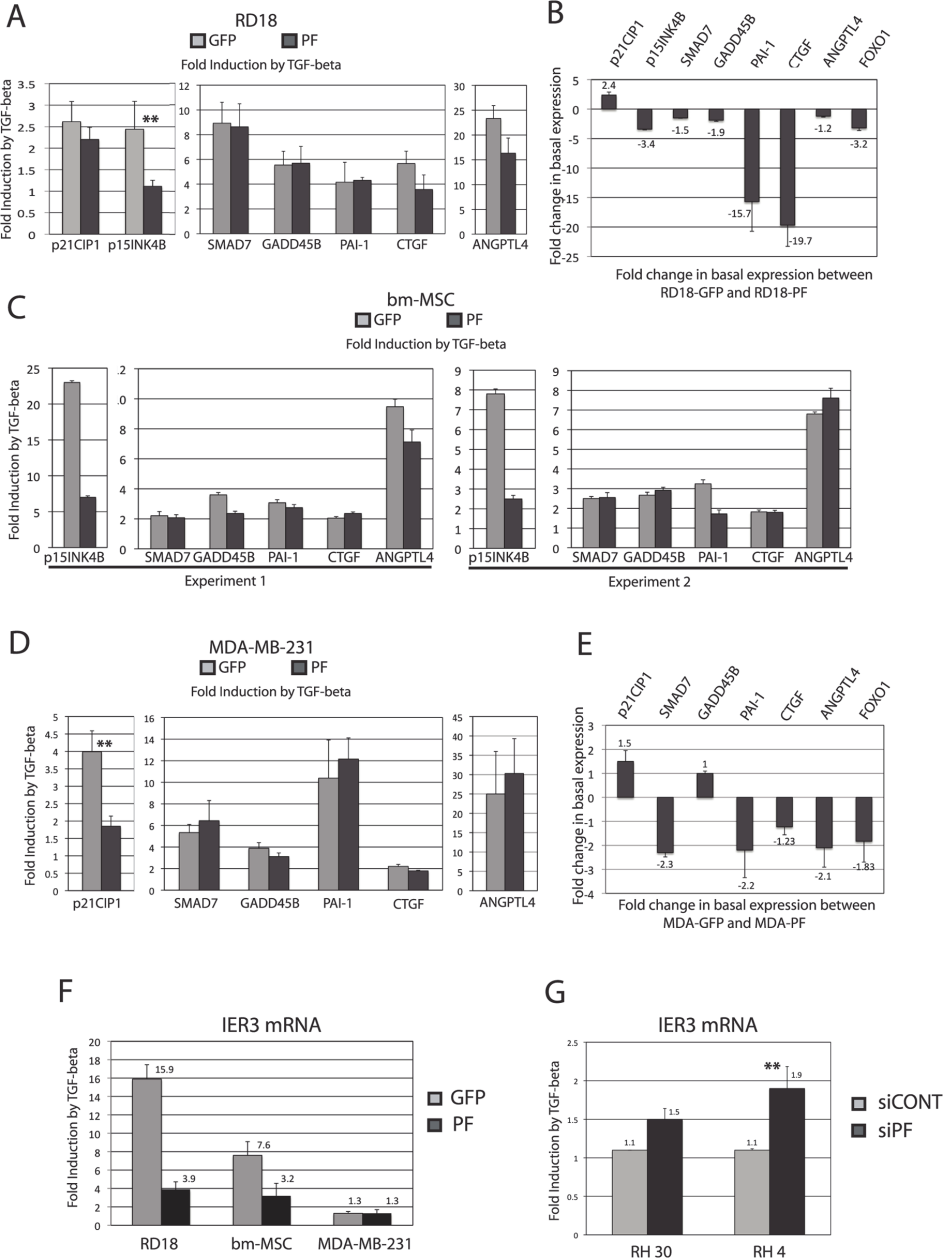
Ectopic expression of PAX3-FOXO1 interferes with the transcriptional response to TGF-β and affects basal level of expression of FOXO-regulated genes. **(A, C, D and F)** Indicated cell types were infected with lentiviruses carrying either PAX3-FOXO1 (PF) or GFP coding sequences. Pools of cells were checked for PF or GFP expression and were challenged with TGF-β for 5 hours. Cells were lysed, RNA extracted and levels of indicated mRNAs were measure by real-time PCR and plotted as fold induction by TGF-β. (**A)** Represents the mean of four experiments; **P<0.01 in two-tailed Student test. (**C)** Two independent infections and TGF-β inductions, in experiment 1 cells are in MSC medium (Lonza), in experiment 2 in DMEM. (**D)** Represents the mean of three experiments, **P<0.01 in two-tailed Student test. (**F)** Represents the means of two experiments. **(B** and **E)** the effect that PAX3-FOXO1 exerts on the basal level of expression of the indicated genes is represented as fold induction or repression (if value is negative) compared with the expression level found in GFP-control infected cells. **(G)** RH30 and RH4 cells were transfected with a control siRNA (siCON) or a siRNA targeting PAX3-FOXO1 (siPF), 32 hours post-transfection cells were challenged with TGF-β for 16 hours and IER3 mRNA levels were measured.

As a complement for the loss of function experiments performed in the ARMS cell lines (Fig. 2), gain of function experiments were performed by infecting various cell lines with lentiviruses carrying PAX3-FOXO1-encoding (PF) or GFP-encoding sequences (as a control). GFP and PF-infected cell pools respectively showed very high percentages of GFP-positive cells (above 90%) and sustained expression of *PAX3-FOXO1* mRNA and protein as determined by real-time analysis (S2 Table) and western blot (Fig. 2B lane 5 and 6 and S3 Fig.). Fig. 5 A and B show the results obtained following the infection of RD18 ERMS cells. The strongest and most convincing transcriptional response to TGF-β was seen with *p15INK4b*, which showed an almost complete loss of response in all experiments. Some reduction in TGF-β-response was observed with other genes, such as *p21CIP* and *CTGF*, but the effects were not strong enough to be convincing. The gain of function experiment also produced important effects on basal gene-expression (Fig. 5 B), which are reminiscent of the results obtained in the PAX3-FOXO1-loss of function experiment. Good complementarity with the loss of function experiment was shown (*p15INK4B*, *FOXO1* and *PAI-1* expression were repressed in the gain of function experiment and induced in the loss of function experiment).

The PAX3-FOXO1 effect was also tested on primary cell cultures. Fig. 5C shows the results obtained in two independently infected pools of primary human bone marrow-derived Mesenchymal Stem Cells (bm-MSC). The GFP- infected cells showed a significant increase in p15INK4B expression following TGF-β treatment. This increase strongly diminishes following PAX3-FOXO1 expression (Fig. 5C), once more confirming this gene's particular sensitivity to PAX3-FOXO1 expression. Basal level expression is less affected by PAX3-FOXO1 expression than it was in RD18 cells (S1A Fig.). *p21CIP* expression is not induced but slightly repressed by TGF-β in bm-MSC-GFP, and PAX3-FOXO1 expression has no effect on this regulation (S1B Fig.).

Fig. 5D shows the results obtained in a non-rhabdomyosarcoma cancer-derived cell line. In mammary carcinoma MDA-MB-231 cells, PAX3-FOXO1 expression consistently reduces the induction of p21CIP by TGF-β while the response of other genes remained unchanged. The influence of TGF-β could not be evaluated on *p15INK4B* because it is not expressed in this cell line at all. The effect that PAX3-FOXO1 has on basal expression is shown in panel E.

In the study on keratinocytes by Gomis et al. [13], a total of 11 genes were found to be regulated by TGF-β in a FOXO-dependent manner. So far this study on Rhabdomyosarcoma has investigated 5 of them (p21CIP1, p15INK4B, CTGF, GADD45B and SGK). It was therefore decided to analyze the remaining ones (GADD45A, IER3, JAG1, LEMD3, CDC42EP3 and OVOL1). Of these, IER3 was strongly induced by TGF-β in RD18 and in bm-MSC primary cells (Fig. 5 F). In both cases, the ectopic expression of PAX3-FOXO1 strongly compromised this induction (Fig. 5 F). Fig. 4G shows the result of the PAX3-FOXO1 loss of function experiment. The reproducible recovery of TGF-β-inducibility was observed in RH4 cells while a weaker, but non-statistically significant recovery was observed in RH30 cells. The overall behavior of the IER3 gene strongly supports our hypothesis that PAX3-FOXO1 can interfere with the FOXO-dependent TGF-β response.

## Discussion

FOXO family members are essential mediators of the transcriptional response to PI3K-AKT signaling. A mutation of the human FOXO ortholog, daf-16, in C.elegans reverts the lifetime extension caused by mutations in the insulin receptor or in PI3K, which highlights its crucial role in these pathways. In mammals, many components of the PI3K-AKT pathway show frequent alterations in cancer [35]. AKT is a main regulator of FOXO activity [34] and inhibits FOXO nuclear function via phosphorylation-dependent re-localization to the cytoplasm. This inhibition of FOXO nuclear function is part of the mechanism by which PI3K-activating mutations, or PTEN inhibitory mutations, operate. For example, AKT-mediated phosphorylation and the nuclear exclusion of FOXO are required for Myc-induced proliferation and transformation to occur [36,37]. Evidence of FOXOs' direct involvement in tumorigenesis has been concealed by the fact that FOXO1, FOXO3 and FOXO4 are redundant. Their tumor-suppressive activity has been uncovered by their simultaneous disruption in mice [17] or the use of dominant-negative mutants [37]. We herein describe a natural-occurring mechanism by which FOXO activity is inhibited or disturbed; the expression of the chimeric PAX3-FOXO1 protein found in ARMS. We show that a reduction in PAX3-FOXO1 expression in ARMS cells, or its ectopic expression in non-ARMS cells, affects the basal expression of FOXO target genes. We show that PAX3-FOXO1 is able to inhibit FOXO-dependent transcription in transactivation experiments. FOXOs participate in the TGF-β response [11,13], and we show that PAX3-FOXO1 interferes with the FOXO-dependent TGF-β response: *p15INK4B*-inducibility by TGF-β, known to be dependent on FOXO activity [12], is restored when PAX3-FOXO1 expression is inhibited in ARMS cells. In the complementary gain of function experiment, the ectopic expression of PAX3-FOXO1 inhibits *p15INK4B*- and *p21CIP*-inducibility by TGF-β in cell line and primary cultures. We show, using transactivation experiments, that PAX3-FOXO1 is able to inhibit the transcriptional response to TGF-β on a bipartite FOXO-SMAD response element.

Unlike FOXOs, PAX3-FOXO1 remains in the nucleus independently of AKT activity [38]. This characteristic gives PAX3-FOXO1 a clear advantage in interfering with FOXO nuclear-activity as it will be present in any circumstance, while FOXO levels will fluctuate due to nucleo -cytoplasmic shuttling. Our transactivation experiments point toward the possibility that PAX3-FOXO1 may exert direct action on the promoter of the target genes where it may disturb normal FOXO action and, via a similar mechanism, disturb the FOXO-dependent TGF-β response. We cannot however exclude an indirect effect where PAX3-FOXO1-regulated proteins interfere with FOXO and FOXO-dependent TGF-β pathways.

In light of what we know about FOXOs, how can PAX3-FOXO1 directly interfere with FOXO function? PAX3-FOXO1 is missing an important part of the Forkhead DNA-binding domain and is therefore not thought to possess FOXO-DNA binding activity [39]. This lack of DNA-binding would appear to exclude the possibility of a dominant-negative mechanism via the non-functional-occupancy of FOXO binding sites. Furthermore, FOXOs are known to act as monomers, excluding a possible dominant-negative mechanism via non-functional hetero-dimerization between FOXOs and PAX3-FOXO1. It may be that PAX3-FOXO1 and FOXOs take part however in a common functional complex via which the observed functional interference is mediated. Alternatively, PAX3-FOXO1 might squelch transcription by competing away co-activators. This type of mechanism has been observed in *in vitro* experiments when elevated transcription factor expression is reached but has also been proposed to occur *in vivo* as a normal means of gene regulation [40]. The “*in vitro*” transforming activity of PAX3-FOXO1 has been shown to be very sensitive to concentration [41]. The squelching mechanism is, by definition, concentration dependent [40]; expression that is too low may have no effect and expression that is too high may induce counter-productive effects. Therefore, only a very narrow range is effective in achieving a given biological effect in a given cellular context. The cellular context sets the presence and the concentration of the squelching-partners, which will determine the functional outcome of PAX3-FOXO1's presence. The stringent conditions that may be required for biological activity could be the reason why it has been difficult to obtain tumors in mouse models expressing ectopic PAX3-FOXO1 [8,42,43], and why we observe partial and cell type-specific effects in our ectopic-expression of PAX3-FOXO1. The partial effect seen by PAX3-FOXO1 in RD18 cells on the TGF-β-transcription response of *p21CIP* and *p15INK4B*, for example, was confirmed by the lack of a functional effect on the growth-inhibitory action of TGF-β in this cell line (data not shown). We feel that understanding the reason for this incomplete action might reveal important features of PAX3-FOXO1's mode of action and we are currently investigating it. The exceptional complementarity between the gain of function and loss of function experiments is, however, striking. If we confront the results obtained on basal gene expression in RD18 gain of function and RH30 loss of function experiments, perfect complementarity is observed (increase in loss of function and decrease in gain of function) with the exception of *p21CIP*. This complementarity strongly sustains the validity of the individual experiments. In the RH4 loss of function experiment, three outliers are observed which may reflect differences in the transcription factor and/or interacting protein makeup of the cell. For example, we noticed that RD18 and RH30 cells express *PAX3* mRNA, while RH4 cells do not (S2 Table). This has also been reported in the literature [44]. PAX3 forms homodimers through homeodomain-interactions and could potentially affect PAX3-FOXO1 function by binding to it. This could be the reason for the cell type specific differences between RH4 and RH30 that we have observed.

The alteration of the TGF-β-transcriptional response by PAX3-FOXO1 is gene-specific. This may be due to the specific arrangement of the FOXO and SMAD DNA-binding sites in the different gene promoters. In fact, it is the genes that possess a bipartite SMAD-FOXO1 binding element that undergo disturbed TGF-β-inducibility (in loss of function as well as gain of function experiments). TGF-β is known to possess a dual and antagonistic action on tumor growth; tumor suppressing activity (via its growth arrest and pro-apoptotic functions) and pro-tumor activity at the same time (via Epithelial-to-Mesenchymal Transition induction which favors tumor spread). One could imagine that PAX3-FOXO1’s role in tumorigenesis is to inhibit the first action of TGF-β (tumor suppressor) while preserving the second (pro-invasive). Our data are compatible with such a mechanism. It may require the identification of ARMS' cell of origin, whose identity is still under debate [20,21], before it is possible to prove such a mechanism. Regarding the ERMS cell line, we found that the RD18 cells are remarkably good responders to TGF-β in terms of transcriptional response and growth arrest, with the latter being previously reported in the parental RD cell line [19]. We also confirm that the transcriptional effects seen upon the TGF-β treatment of RD18 cells are also seen with RD cells (see S2 Fig.). It would be interesting to know whether this is a general characteristic of ERMS cells or rather a particular phenotype of RD cells.

ARMS cells lose viability [22,23] upon the lowering of PAX3-FOXO1 levels by RNAi, and we can confirm this observation here: three days after transfection, siFP transfected cultures show visibly fewer cells then control siCONT-transfected ones. The present finding on the capacity of PAX3-FOXO1 to interfere with FOXO activity supports the hypothesis that the loss of viability observed upon PAX3-FOXO1 expression suppression is due to the recovery of FOXO activity. We are currently investigating this possibility. Pro-apoptotic and/or growth suppressive FOXO activity is well documented and characteristic for tumor suppressive capacities.

As mentioned above, FOXO1, 3 and 4 are redundant for tumor suppressor activity. We show that the capacity of PAX3-FOXO1 to inhibit FOXO1-nuclear transactivation activity extends to FOXO3 and FOXO4, but also to their constitutive -active non AKT-sensitive mutant versions. This result strongly sustains the hypothesis that FOXO tumor suppressor activity loss might be decisive in the generation of alveolar Rhabdomyosarcoma. This conclusion is also strongly supported by the concordance in data shared with So and Cleary [45]. They reported that MLL-FOXO4 (a chimeric protein product of a genomic translocation that arises in a small number of acute leukemia cases) was able to suppress FOXO3-mediated apoptosis in Ba/F3 cells as well as FOXO3 -mediated transcriptional activation. The characteristics of the FOXO4 moiety in MLL-FOX4 are very similar to those of the FOXO1-moiety in PAX3-FOXO1 (truncation Forkhead domain with loss of DNA-binding activity).

We show that PAX3 expression has no inhibitory effect on FOXO activity. It is therefore likely that the FOXO-interference activity of PAX3-FOXO1 is due to the FOXO1 moiety of PAX3-FOXO1. Non-classical gene-regulation exerted by a non-DNA-binding FOXO mutant has been described [46,47,48]. These surprising effects were shown on genes without FOXO DNA-binding elements (IRS) in their promoter and were explained by FOXO's capacity (and also mutant FOXO lacking DNA-binding capacity) to bind gene promoters indirectly by making complexes with other DNA-binding proteins targeted to these promoters. Our results differ in that we see an IRS-dependent effect of a non-IRS-binding mutant. In their study, Ramaswamy et al. [46] performed global gene expression analyses on an over-expressed mutant FOXO which lacked DNA-binding activity. Their study should also have picked-up IRS-dependent effects, but did not. A logical explanation for this apparent discrepancy exists, however.

The Ramaswamy et al. study was performed using PTEN null cells in which endogenous nuclear FOXO activity is at minimum. Therefore, it is highly probable that no interference with endogenous IRS-dependent FOXO activity was seen in their experiments because no initial nuclear FOXO activity was present.

Another aspect to be considered is myogenic differentiation, whose obstruction may contribute to tumorigenesis. The inhibition of FOXO activity by PAX3-FOXO1 might limit the capacity of the cells to fully differentiate; FOXO1 has been shown to be required in the process [49]. TGF-β, on the other hand, has been shown to inhibit myogenic differentiation in normal muscle [50], and also in Rhabdomyosarcoma [51,52]. The inhibition of the TGF-β pathway by PAX3-FOXO1, described here, may represent an apparent contradiction. However, we would again like to emphasize that the mechanism we unveil here is the specific inhibition of (or interference in) the part of the TGF-β response which is dependent on FOXO. It would therefore be interesting to see whether the TGF-β pathway component that participates in myogenic differentiation inhibition is still active in ARMS cells. TGF-β had no apparent effect on differentiation in our experiments and also in the publication by Ye et al. [19], but it has to be said that cells were not subjected to differentiation-inducing conditions to test possible differentiation effects.

The present study unveils a new mechanism of action for PAX3-FOXO1. Knowledge of it may provide important clinical impact: interference of FOXO-activity and FOXO-dependent TGF-β pathways may be a target and its relief a goal for further therapies. Further work on defining the *in vivo* contribution of this mechanism to tumor formation is, however, essential.

## Materials and Methods

### Cell lines, viability test and Luciferase assay

The ERMS cell line RD18 [18] and the ARMS cell lines RH30 and RH4 [53] were a kind gift from the laboratory of Prof. Pier-Luigi Lollini (University of Bologna). As described throughout this work RH30 and RH4 cells express the translocation product PAX3-FOXO1 (summarized in S2 Table), as well as MyoD1 and Myogenin (MYOG) (S2 Table), this authenticating their origin. RD18 express PAX3, MYOD1 and MYOG (S2 Table), this reflecting their myogenic origin. 293T and MDA-MB-231 cells were from ATCC. RD cells were from Riccardo Taulli. All cells were cultured in DMEM high glucose with 10% fetal bovine serum supplemented with Penicillin and Streptomycin and L-Glutamine (Gibco). Bone marrow-derived Mesenchymal Stem Cells (bm-MSC) were obtained from Lonza (Basel, Switzerland) and cultured in mesenchymal stem cells basal medium (MSCBM, Lonza) or DMEM as indicated in legend of Fig. 3. Human Recombinant TGF-β 1 was from R&D systems. Viability test was performed with the MTT colorimetric kit (Millipore) according to the manufacturer’s instructions. For luciferase assays, plasmid DNA were transfected using lipofectamine 2000 (invitrogen). Extracts were prepared 48 hours or 72 hours after transfection using the Dual-Glo luciferase assay system or the steady-Glo assay system (Promega). When Dual-Glo was used the RLTK promoter-driven renilla luciferase gene was the internal control for transfection efficiency. Luciferase activity was measured on a GloMax multidetection plate reader (Promega). Western blots confirmed transient expression of PAX3-FOXO1 and FOXO family members. In general FOXO1 expression seemed higher than PAX3-FOXO1 expression. However, it could be that the antibody (directed against the C-terminal part of FOXO1) recognizes more total epitopes on wild type FOXO1 compared to PAX3-FOXO1 and therefore give a higher signal for FOXO1 on western blots, this precluding adequate quantification.

### Plasmids, siRNA transfection, lentiviral vectors and viral infection

The human PAX3-FOXO1 sequence was cut from pBabe-PAX3-FOXO1 (a kind gift of Dr. B.W. Schäfer, University Children’s Hospital, Zurich, Switzerland) by BamH1-Sal1 digestion and introduced in the BamH1-XhoI sites of pcDNA3. Human SMAD expression vectors were a kind gift of Dr. Aristidis Moustakas (LICR, Uppsala, Sweden). SBE4-luc was the kind gift of Dr. Bert Vogelstein (John Hopkins Oncology Center, Baltimore). pcDNA3-Flag-FKHR and 3XIRS-luc was the kind gift of Dr. Eric D. Tang (University of Michigan Medical School). 4XSFRE-pGL2 was obtained by PCR from a 2XSFRE-pGL2 construct with the primers 4XSBR-Synt and pGL2-rev. The PCR product was digested by Nhe-1 and ligated into pGL2-luc. For the generation of 2xSFRE-pGL2 promoter the oligos 2xSFRE-upper and 2xSFRE-lower were annealed and cloned in the Nhe/Bgl2 cleaved pGL2-promoter vector. Expression vectors for FOXO1-AAA (Kunliang Guan), FOXO3-AAA (Michael Greenberg), FOXO4 (Domenico Accili) and mouse Pax3 (Jonathan Epstein) were purchased from Addgene (Cambridge, USA). Empty pcDNA3 plasmid (Invitrogen) was used as transfection carrier.

The siRNA siPF sequence was reported previously [22] and is displayed in S1 Table with the sequence of the control siRNA used (siCON). siRNAs were synthesized by Eurofins MWG Operon-Biotech AG, Ebersberg, Germany. siRNAs were transfected using lipofectomine (invitrogen) following the indication of the manufacturer. The day of transfection cells were at a density of 30 to 40%. Fresh medium was given 16 hours after transfection. Experiments (TGF-β inductions and sample collection) were performed between 30 to 48 hours after transfection avoiding important loss of viability observed at later times.

For lentiviral expression of PAX3-FOXO1, the GFP cDNA sequence was excised from pCCLsin.PPT.hPGK.GFP vector and replaced with PAX3-FOXO1 cDNA. For the production of lentiviruses, 293T were tansfected with a combination of pCCLsin.PPT.hPGK.PAX3-FOXO1 or pCCLsin.PPT.hPGK.GFP and packaging plasmids pVSVG, pMDL and pREV using fugene 6 (promega). Viral supernatants were harvested over 36 to 72 hours, filtrated (0.22 μm pore) and used to infect cultures of subconfluent recipient cells in the presence of 8μg/ml polybrene (sigma). The viral supernatant represented half of the total culture medium and was left on recipient cells for 15 to 24 hours. Experiments involving infected RD18 and MDA-MB-231 were performed several days to weeks after infection. For infected bm-MSCs the experiments were performed in the range of 4 to 6 days after infection while cells looked nice and vital. GFP as well as Pax3-FOXO1-expressing bm-MSCs lost viability when kept more than10 days in culture after infection.

### Gene expression studies

Total RNA was extracted with TRI reagent (Applied Biosystems) according to the manufacturer's instructions. First-strand cDNA was produced using High Capacity cDNA Reverse Transcription Kit (Applied Biosystems).

Real-time PCR experiments were performed in 20 μl reaction mixture with Power SYBR Green PCR Master Mix (Applied Biosystems) using a 48-well StepOne real-time PCR System (Applied Biosystems). The sequence-specific oligonucleotide primers were selected with the use of the “Primer Express” software (Applied Biosystems) and were purchased from Eurofins MWG Operon-Biotech AG, Ebersberg, Germany. The primer sequences are listed in S1 Table. For p15INK4B two sets of independent primers were used for confirmation, as the first set lost some specificity in case of low expression (both sets gave equivalent results). Thermal cycling conditions were as follows: a 95°C for 10 min followed by 40 cycles of amplification at 95°C for 15 s and 60°C for 1 min. For all real-time PCR analyses, *β-ACTIN* or *TBP* mRNAs were used to normalize mRNA inputs and gave equivalent results. Fold change expression with respect to control was calculated for all samples.

### Western blot and protein analysis

Protein extract were made using RIPA buffer containing protease and phosphatase inhibitor mixes (SIGMA). After protein electrophoresis, the gel was blotted on PDF membrane using. Anti-FKHR (H-128) and Anti-Actin antibodies were from Santa Cruz Biotechnology, INC., Anti phospho-Smad2 (#3101) was from Cell Signaling Technology.

## Supporting Information

S1 Fig Ectopic expression of PAX3-FOXO1 in bm-MSC. **A**: the effect that PAX3-FOXO1 (PF) exerts on the basal level of expression of the indicated genes is represented as fold induction or repression (if value is negative) compared with the expression level found in GFP-control infected cells. **B**. Pools of cells expressing PF or GFP were challenged with TGF-β for 5 hours. mRNAs levels were measure by real-time PCR and plotted as fold induction by TGF-β.
(EPS)

S2 Fig TGF-β response of RD cells is similar to the one observed in RD18 cells. Induction of indicated mRNAs following 5 hours treatment with 5ng/ml TGF-β. mRNAs levels were measured by real-time PCR and plotted as fold induction.
(EPS)

S3 Fig PAX3-FOXO1 protein and FOXO1 protein expression levels in the indicated cell lines. Levels of PAX3-FOXO1 and FOXO1 protein were evaluated by western blot using an anti-FOXO1 antibody recognizing the C-terminal part of the FOXO1 protein present in PAX3-FOXO1. MDA-MB-231 and RD18 were stably infected with GFP or PAX3-FOXO1 (PF) expressing lentivirus. As a control for PAX3-FOXO1 and FOXO1, extracts from 293T transiently transfected with FOXO1 or PAX3-FOXO1 expressing plasmids cells were included (lane 7 and 8). All lanes were loaded with the same amount of total proteins with the exception of lane 7 and 8.
(EPS)

S1 Table Oligonucleotides used in the study. (PDF)

S2 Table Relative basal level of Expression of mRNAs of Interest. Relative expression of indicated mRNAs determined by Real-Time PCR: ++++++: (C_T_: 20–21), +++++: (C_T_: 22–23),++++: (C_T_: 24–25), +++: (C_T_: 26–27), ++: (C_T_:28–29), +: (C_T_: 30–32),—: (no expression or c_t_ >35). nd: not determined. C_T_ is the threshold cycle
(PDF)
