# Supplementary material for: The PAX3-FOXO1 Fusion Protein Present in Rhabdomyosarcoma Interferes with Normal FOXO Activity and the TGF-β Pathway
Source: PLoS One. 2015 Mar 25;10(3):e0121474. doi: 10.1371/journal.pone.0121474 (PMC4373809; doi:10.1371/journal.pone.0121474)
Supplement: S1 Table — (PDF) [file pone.0121474.s004.pdf]

	For cloning
2XSFRE-upper	5'-CTAGCCCAAAGTAAACAGACAGACAATGTCTAGTCTATTTCCC AAAGTAAACAGACAGACAATGTCTAGTCTATTTA-3'
2XSFRE-lower	5'-GATCTAAATAGACTAGACATTGTCTGTCTGTTTACTTTGGGAAAT AGACTAGACATTGTCTGTCTGTTTACTTTGGG-3'
4XSFRE-Synt	5'-CGCGCTAGCCCAAAGTAAACAGACAGACAATGTCTAGTCTATTTCC CAAAGTAAACAGACAGACAATGTCTAGTCTATTTC-3'
pGL2-rev	5'-CTTTATGTTTTTGGCGTCTTCCA-3'
	**For Real-Time PCR**
BETA-ACTIN<br>ACTB<br>ID: 60	F: 5'-TGAAGATCAAGATCATTGCTCCTC-3'<br>R: 5'-CACATCTGCTGGAAGGTGGAC-3'
TBP<br>ID: 6908	F: 5'-TGTGCACAGGAGCCAAGAGT-3'<br>R: 5'-ATTTTCTTGCTGCCAGTCTGG-3'
P21CIP1<br>(CDKN1A)<br>ID: 1026	F: 5'-GAGACTCTCAGGGTCGAAAACG-3'<br>R: 5'-ATTAGGGCTTCCTCTTGGAGAAG-3'
SMAD7<br>ID: 4092	F: 5'-CCTTAGCCGACTCTGCGAACTA-3'<br>R: 5'-CCAGATAATTCGTTCCCCCTGT-3'
PAI-1<br>(SERPINE1)<br>ID: 5054	F: 5'-CCTCATCCACAGCTGTCATAGTCT-3'<br>R: 5'-GGCCCATGAAAAGGACTGTTC-3'
PAX3-FOXO1	F: 5'-AATGGCCTCTCACCTCAGAATTC-3'<br>R: 5'-CTTGCCACCCTCTGGATTGA-3'
P15INK4B<br>(CDKN2B)<br>ID: 1030	Primer 1<br>F: 5'-AGTGGAGAAGGTGCGACAGCT-3'<br>R: 5'-CCATCATCATGACCTGGATCG-3'<br>Primer 2<br>F: 5'-CCAACGGAGTCAACCGTTTC-3'<br>R: 5'-CCCATCATCATGACCTGGATC-3'
CTGF<br>ID: 1490	F: 5'-AAGAGAACATTAAGAAGGGCAAAAAG-3'<br>R: 5'-CAGCCAGAAAGCTCAAACTTGA-3'
ANGPTL4<br>ID: 51129	F: 5'-GGCCTCTCCGTACCCTTCTC-3'<br>R: 5'-GGCCGTTGAGGTTGGAAT-3'
GADD45B<br>ID: 4616	F: 5'-CGGTGGAGGAGCTTTTGGT-3'<br>R: 3'-GCTGTCTGGGTCCACATTCA-3'
FOXO1<br>ID: 2308	F: 5'-ATGGTCAAGAGCGTGCCCTA-3'<br>R: 5'-TGTGTAGGGACAGATTATGACGAATT-3'
SGK1<br>ID: 6446	F: 5'-GCATGCAAACACCCTGAAGTT-3'<br>R: 5'-GAAGGGTTGGCATTCATAAGCT-3'
PAX3<br>ID: 5077	F: 5'-AACCCCACCATTGGCAATG-3'<br>R: 5'-CACCGTGGTTGGTCAGGAGT-3'
IER3<br>ID: 8870	F: 5'-CCGCAGGGTTCTCTACCC-3'<br>R: 5'-GGATCTGGCAGAAGACGATG-3'
MYOD1<br>ID: 4654	F: 5'-TGCCACAACGGACGACTTC-3'<br>R: 5'-CGGGTCCAGGTCTTCGAA-3'

MYOG<br>ID: 4656	F: 5'-GCTCACGGCTGACCCTACA-3'<br>R: 5'-AGACACATCTTCCACTGTGATGCT-3'
	**For siRNA**
siCON	Sense: 5'-CUACUAUACCGAUACUCCCTT-3'<br>Antisense: 5'-GGGAGUAUCGGUAUAGUAGTT-3'
siPF	Sense: 5'-CCUCUCACCUCAGAAUUCATT-3'<br>Antisense: 5'-UGAAUUCUGAGGUGAGAGGTT-3'
