# Supplementary material for: The PAX3-FOXO1 Fusion Protein Present in Rhabdomyosarcoma Interferes with Normal FOXO Activity and the TGF-β Pathway
Source: PLoS One. 2015 Mar 25;10(3):e0121474. doi: 10.1371/journal.pone.0121474 (PMC4373809; doi:10.1371/journal.pone.0121474)
Supplement: S2 Table — Relative expression of indicated mRNAs determined by Real-Time PCR: ++++++: (CT: 20–21), +++++: (CT: 22–23),++++: (CT: 24–25), +++: (CT: 26–27), ++: (CT:28–29), +: (CT: 30–32),—: (no expression or ct >35). nd: not determined. CT is the threshold cycle (PDF) [file pone.0121474.s005.pdf]

mRNAs / Cells	PAX3	PAX3-FOXO1	FOXO1	TBP	MYOD	MYOG
RD18	+++	-	++	++++	+++++	+++
RH30	++++	++++	+++	++++	++++	++++
RH4	-	++++	++++	++++	++++	++++
RD18-GFP	+++	-	++	++++	+++++	+++
RD18-PF	++++	++++	+	++++	++++++	++++
Bm-MSC-GFP	-	-	nd	+++	+	+
Bm-MSC-PF	-	++++	nd	+++	+++	+
MDA-DB-231-GFP	-	-	nd	++++	+	+
MDA-DB-231-PF	-	++++	nd	++++	+	+
